# Perception of risk of HIV infections and sexual behaviour of the sexually active university students in Zimbabwe

**DOI:** 10.1080/17290376.2014.886082

**Published:** 2014-06-12

**Authors:** Njabulo Nkomazana, Pranitha Maharaj

**Affiliations:** ^a^ PhD Candidate at the School of Built Environment and Development Studies, University of KwaZulu-Natal, Durban, Republic of South Africa; ^b^ PhD, is an Associate Professor at the School of Built Environment and Development Studies, University of KwaZulu-Natal, Durban, Republic of South Africa

**Keywords:** university students, HIV risk perceptions, sexual behaviour, Zimbabwe, étudiants de l'université, risque d'infection à VIH, comportement sexuel, Zimbabwe

## Abstract

The study sought to establish university students' perceptions of risk of HIV infections. A cross-sectional survey was conducted on 345 sexually active students at two universities in Zimbabwe (one state and one private). Results revealed that above a quarter of the respondents felt at risk of getting HIV due to their regular partners' sexual behaviours and more than half felt at risk of getting HIV due to their casual partners' sexual behaviours. In addition, a third of the respondents acknowledged the HIV risk due to their own sexual behaviours. More state university respondents felt exposed to HIV infections due to own sexual behaviours than their private university counterparts. Despite these revelations, only 66.56% had earlier thought of their chances of getting infected with HIV. Personal HIV risk perceptions were low, reported by 27.76% of the sexually active respondents. Almost all respondents described their fellows' sexual behaviours as either risky or very risky.

## Résumé

L'étude visait à évaluer la perception du risque d'infection à VIH des étudiants de l'université. Une 'étude transversale a été menée sur 345 étudiants sexuellement actifs dans deux universités du Zimbabwe (une étatique et une privée). Les résultats ont révélé que plus d'un quart des répondants s'estimaient être exposés au risque de contracter le VIH à cause du comportement sexuel de leurs partenaires réguliers et plus de la moitié se sentait exposer au risque de contracter le VIH à cause du comportement sexuel de leurs partenaires occasionnels. En outre, un tiers des répondants ont reconnu qu'ils étaient exposés au risque de contracter le VIH à cause de leur propre comportement sexuel. Il y a eu plus des répondants de l'université de l'État qui ont estimé qu'ils étaient exposés au risque d'infection à VIH à cause de leur comportement sexuel que leurs homologues de l'université privée. Malgré ces affirmations, seulement 66,56% s'étaient déjà imaginé qu'ils étaient exposés au risque d'être infecté par le VIH. La perception de contracter le VIH au niveau individuel était faible, c'était reconnu par 27,76% des répondants sexuellement actifs. Presque tous les répondants décrivent le comportement sexuel de leurs semblables comme étant à risque ou très à risque.

## Introduction

Psychosocial theories of behaviour change, such as the Health Belief Model, the AIDS Risk Reduction Model, the Trans-theoretical Model (also known as the stages of change model) and the Theory of Reasoned Action, stress the pivotal role played by an individual's perception of the risk of a given problem in the process of human behaviour change (Catania, Kegeles & Coates 1990; Fishbein, Middlestadt & Hitchcock 1994; Prochaska, DiClemente & Norcross 1992; Rosenstock, Strecher & Becker 1994). These theories underscore that a person's recognition of his/her susceptibility to any problem or disease, including HIV, is vital in prompting the person to take actions that may reduce his/her vulnerability. In other words, the actions that an individual takes to protect himself/herself from any disease, including HIV, will to a large extent depend on his/her perception of the risk of the disease. In the context of HIV, an individual's perception of the risk of HIV infections often affects a number of factors such as whether or not he/she has a HIV test or uses condoms during sexual acts. In spite of these propositions, the nexus between sexual behaviour and perceived risk of HIV infection remains poorly appreciated (Akwara, Madise & Hinde 2003).

While the entire spectrum of young people is heavily affected by the HIV scourge, the degree of the severity of their problem may vary depending on the cultural, socio-demographic, political and economic environments that they find themselves in. This study concentrates on one group of young people at high risk of HIV infections, university students. Worldwide, this section of young people has of late deservedly received research attention due to the different circumstantial risks that they face. It is often argued that although the majority of university students are part of the 15–24 age group, due to their life circumstances their vulnerability to HIV while at university is uniquely high (Masvaure, Terry, Adlis & Mhloyi 2009). Generally, the environment at universities is sexually permissive, since it is where young people from different backgrounds and sexual orientations meet and live together, often with little or no parental and administrative prohibitions (Masvaure *et al*. 2009; Omungo 2008; Sabone, Ntsayagae, Brown, Seboni, Mogobe & Sebego 2007). Research suggests that universities are high-HIV-risk institutions for the transmission of HIV infections because of the predominance of risky sexual activities (Njagi & Maharaj 2006). In fact, the university environment, if not properly managed, can provide a very fertile ground for risky sexual behaviours and the spread of new HIV infections (Adefuye, Abiona, Balogun & Lukobo-Durrell 2009).

Studies suggest that despite possessing high levels of knowledge on HIV and AIDS, many university students continue to engage in risky sexual practices and appear not personally concerned about contracting the virus (Ferrer, Cianelli, Guzman, Cabieses, Irarrazabal, Bernales, *et al*. 2007; Ntata, Muula, Siziya & Kayambazinthu 2008; Peltzer 2005; Rahamfey, Rivard, Ravaoarinoro, Ranaivoharisoa, Rasamindrakotroka & Morisset 2008). As such, several studies find personal HIV risk perception of university students to be very low. This practice is usually referred to as ‘denial and distancing’ (Brown, Outlaw & Simpson 2000). Low personal HIV risk perception is very dangerous as it may fuel the spread of HIV infections, since students may continue to lead risky sexual lives as they think that they are not at risk of the virus. For instance, a South African study revealed that the majority of students (83%) had never been worried that they could contract HIV, with only 26% feeling that their lives were at risk of contracting HIV infections (Peltzer 2005). However, other studies reveal that some university students may engage in risky sexual practices with full knowledge of their personal health risks, yet fail to change their sexual behaviours (Maswanya, Moji, Horiguchi, Nagata, Aoyagi, Honda, *et al*. 1999; Nshindano & Maharaj 2008; Peltzer, Nzewi & Mohan 2004). This underscores that some students may at times feel helpless in adopting preventative measures to reduce their health risks. Even if they realised their exposures to health hazards, they sometimes deliberately chose to ignore it, thereby adopting fatalistic attitudes. The failure to translate intention to avoid risky sexual behaviours into practical actions that reduce the risk of HIV infection is due to the fatalistic attitude or lack of self-efficacy (Peltzer *et al*. 2004). The failure to adopt preventative measures in the face of apparent HIV risk is usually common in exploitative relationships and those where money is the ultimate factor driving them.

However, in some studies, personal HIV risk perceptions among university students may be very high (Njikam nee Savage 2005; Omungo 2008). For instance, 75% of students sampled in Cameroon felt that they were at risk of being infected with HIV (Njikam nee Savage 2005). Similarly, a study in Kenya revealed that those who reported consistent use of condoms had high personal HIV risk perceptions, showing that they had already responded to their fears by using condoms consistently (Omungo 2008). These revelations support the assertion that perception of risk of a problem is the primary driver for the adoption of protective actions and may vary from country to country.

Personal HIV risk perception has been a topical issue over the years given its hypothesised association with the adoption of safe sexual behaviours. However, no studies have been carried out on young people in Zimbabwe, to ascertain how it influences the dynamics of the spread of HIV infections. Given the contrasting findings from across Africa and beyond or even from within one country by previous researchers, it becomes apparent that a study on students' personal HIV risk perceptions be carried out in Zimbabwe in order to ascertain how university students perceive their risk of HIV infection and also to establish what influences these perceptions, and in turn how these perceptions affect sexual behaviour. This is in the spirit of establishing the role played by the proximal (the institutional) context in modelling personal HIV risk perception of students in Zimbabwe and also trying to better understand factors behind the spread of HIV infections. This paper was motivated by reports of risky sexual practices by university students in Zimbabwe, despite universal knowledge about HIV and AIDS and availability of many campaigns to reduce the spread of HIV infections.

### The context

The Republic of Zimbabwe is a landlocked Southern African nation sharing borders with South Africa on the south, Botswana on the west, Zambia on the north and Mozambique on the east. The 2002 national census estimated the country's population at 11,632 million, of which approximately 55% are believed to be aged below 15 years and 52% of the population were females (CSO 2002). The majority of people in Zimbabwe (70%) live in rural areas and are dependent on subsistence farming; as such they are often poor. Zimbabwe, like other Southern African countries, is battling to combat one of the most severe HIV epidemics in the world. With the first case of HIV/AIDS reported in 1985 (GOZ 1999; Meursing & Sibindi 2000; Whiteside 1996), the epidemic subsequently spread rapidly throughout the country, and is now believed to be generalised in the country's population. HIV prevalence rates of above 40% have once been reported in some towns and growth points in the country (Pettifor, Straten, Dunbar, Shobiski & Padian 2004; ZMHCW 1994). At the onset of the epidemic HIV prevalence rates were fairly lower in rural areas than they were in urban areas but are now fairly similar, with the 2005–2006 Demographic and Health Survey estimating these rates at 19% and 18%, respectively, for urban and rural areas (CSO & Macro International 2007). Recent statistics show that HIV prevalence in Zimbabwe has been on a steady decline. After reaching a peak of 29.3% in 1998, the HIV prevalence rate fell to 26.5% in 2001, 15.6% in 2007 and eventually to 15% in 2010 (Zimbabwe National Statistics Agency, MEASURE DHS & ICF Macro 2011; ZMHCW 2008). Despite the observed gradual decline over the past decade, HIV prevalence in Zimbabwe remains high and HIV is generalised in the entire population.

The study was conducted among university students from two universities in Zimbabwe (one state-owned and one private-owned). The two universities were purposively chosen. The selection of the two universities was guided by the need to compare and contrast perception of risk of HIV infection and sexual behaviours of university students at the two universities with different administrative values and education curricula.

## Methods

### Data and sampling framework

The data for this study were part of a large study designed to investigate the determinants of risky sexual behaviours among university students in Zimbabwe. It was carried out between April and December 2010. Two universities, one state-owned and one private-owned, were purposively selected for study. In order to maintain anonymity and comply with the recommendations of the Ethical Committee of the private university chosen, the names of the two universities will be withheld. The two universities are hereafter referred to as the ‘state university’ and the ‘private university’. Since the two universities had various modes of entry, only full-time students studying undergraduate bachelor's degrees for a minimum of three years were considered for the present study. This means that only students on conventional degree programmes (day classes) and those on parallel degree programmes (evening classes) were eligible to participate in the study. The study excludes those on part-time basis, such as those studying through block release (visiting school programmes), those studying for diplomas and those doing their postgraduate degrees, since they do not spend much of their time at the university and are also often older, and thus do not fit into the age group of interest, which is 15–24 years. Stratified random sampling was utilised, where students at each university were stratified by their faculties of study and random samples then taken from each faculty. In order to synchronise the faculties found at the two universities, faculties were divided into five strata as follows: Stratum 1 (Commerce or Business), Stratum 2 (Education & Social Sciences), Stratum 3 (Arts & Law), Stratum 4 (Agriculture & Natural Resources Management) and Stratum 5 (Engineering, Science & Technology). A list (or register) of registered students in each university was sought and students were then put into respective strata. Simple random sampling was then used to pick respondents from each stratum, in proportion to the total number of students in their respective strata, using computer-generated random numbers.

Data collection employed a survey approach. The targeted sample size was 890 students drawn from the two universities. The choice of the sample size was informed by guidelines for determining a sample size by Gay, Mills and Airasian (2006), who stated that for any population size of around 1500, 20% should be sampled, whereas for any population greater than 5000, a sample of 400 would be adequate to draw representative conclusions about population parameters (Gay *et al*. 2006). Since the university student population for the state university was 10,231 a sample size of 400 students would have sufficed to produce a representative sample. However, taking into account the inevitable response rate of about 70% often associated with self-administered questionnaires (the main research instrument used to collect data), a sample of 600 students was taken in order to reduce the bias that may be brought about by non-response. More students were selected at first, so that in case of a lower response rate, the final sample size would at least be 400 students for the state-owned university. As for the private university, with a student enrolment estimated at 1482, a sample size of 290 students was taken as this was deemed sufficient to avoid any bias. This was about 20% of students in this university and was thought to produce a representative sample. A self-administered questionnaire was chosen as the most appropriate means of collecting data for this study because of the private and sensitive nature of the questions asked, where honest responses and participation are low when questions are asked face to face. The validly completed questionnaires were 753 (547 from the state university and 206 from the private university respondents), yielding an overall response rates of 89.11% (95.96% for the state university and 74.91% for the private university respondents).

### Measures

Respondents were asked various questions on their risk perceptions of HIV and those of their colleagues. Some questions queried their risk perceptions as a result of their own sexual behaviours, while others queried risk perceptions that resulted from their partners' sexual behaviours (both casual and regular partners). These questions were meant to evaluate respondents' risk perceptions from various sources of HIV risk and also to understand whether or not respondents were familiar with the risks from those sources. The outcome variables in this study were regular partner's fidelity, personal fidelity, HIV risk perception as a result of both regular and casual partners' sexual behaviours, HIV risk perception from own sexual behaviours, chances of getting HIV infections and risk perceptions of fellow students. The majority of questions were directed towards the 345 sexually active respondents in the sample, though two of them were asked to all the respondents in the sample, regardless of their sexual experiences.

Regular partner's fidelity was assessed using the following question: ‘Did your regular partner have any other sexual partner(s) during the time you were in a sexual relationship?’ The answer options to this question were (1) ‘Definitely Yes’, (2) ‘Not Sure’ and (3) ‘Definitely No’. In addition, respondents were asked whether or not they had other sexual partners during the time they were in a sexual relationship with their regular partner, with similar answer options. These questions were meant to assess respondents' awareness to the risk of HIV infections brought about by both their own sexual behaviours and those of their regular partners and also to establish the prevalence of multiple sexual partnerships among university students.

In order to determine students' HIV risk perceptions, respondents were also asked whether or not they felt at risk of contracting HIV, as a result of the sexual behaviours of their regular or casual (non-regular) partners. These questions also had (1) ‘Definitely Yes’, (2) ‘Not Sure’ and (3) ‘Definitely No’ as answer options. This was a direct way to assess students' knowledge of HIV risk exposures posed by both regular and casual sexual relationships. Furthermore, respondents were asked the following question: *Do you feel at risk of contracting HIV due to your own sexual behaviours?* This question measured respondents' risk perception originating from their own sexual behaviours. As a follow-up question, respondents were then queried whether or not they had ever thought about their chances of contracting HIV, as a way to probe their awareness of the HIV risk posed by various sexual behaviours that they come into contact with.

Respondents were also asked to rank their chances of getting HIV infections into the following categories: (1) High; (2) Medium; (3) Low and (4) No chance at all. This question was meant to capture respondents' personal HIV risk perceptions so as to understand whether they knew the risks of HIV posed by their sexual behaviours and those of their sexual partners. Respondents were also asked the following question: *How do you perceive the sexual behaviour of fellow students in the face of HIV?* The answer options to this question were (1) Very safe; (2) Safe; (3) Risky and (4) Very risky. This question was intended to explore how students viewed the sexual behaviour of other students on campus, so as to assess the generally held perceptions about typical university students' sexual behaviours. It was premised on the belief that while some students may be sceptical in describing their own sexual behaviours, possibly due to the social desirability of sexual behaviour, they are comfortable to talk about their fellows' behaviours, which in many instances may actually reflect their own. The last two questions were directed to the total sample regardless of sexual status of the respondents.

After checking for completeness and consistency, the data collected were analysed using STATA software (version 11.0). In the final analysis, the outcome variables were then discussed under gender and place of study categories.

### Ethical considerations

Prior to collecting data, ethical approval for the study was sought and granted by the University of KwaZulu-Natal's Humanities and Social Sciences Ethics Committee. In addition, approval was also granted by the two participating universities through the Pro-Vice Chancellor's Office (for the private university) and through the offices of the Executive Dean of Students and Director of Health Services (for the state university). Making use of the informed consent forms, participants were informed about the purpose of the study and their rights of participation, such as the voluntary nature of participation and confidentiality of collected data. Anonymity of respondents was ensured by removing personal identifiers from the questionnaire. In addition, names of the sampled universities were not made public, as per the condition under which access was granted. In this way, respondents were protected from any adverse consequences that may result from participation in the study.

## Results

Table 1 summarises the characteristics of the 345 sexually active respondents in the sub-sample. Almost 60% of these were males. The majority (69.14%) were in the 21–24 age group, with just 9.2% aged 25 years and above. The mean age of this sub-sample was 21.99 years (standard deviation = 2.04). Only 10.72% of the sexually active respondents were married, with the vast majority, almost 90%, being single. In line with the student populations in the sampled universities, 78.84% of the respondents came from the state university. Most students had spent their first 15 years in urban settings (71.01%) and were in their second year of study (45.22%). About three-quarters of the respondents had had their coital sexual intercourse at the age of 18 or above. Only 8.31% of the respondents had initiated their sexual activities before the age of 15. The median age of coital sex was 19 years.

**Table 1.** Distribution of the sexually active respondents by selected demographic characteristics.

Sample characteristics	*N*	%
*Gender*		
Male	205	59.42
Female	140	40.58
*Age of respondents*		
< 21 years	73	21.66
21–24 years	233	69.14
25+ years	31	9.20
*Marital status*		
Single	308	89.28
Married	37	10.72
*Place of study*		
State university	272	78.84
Private university	73	21.16
*Place of childhood residence*		
Rural	100	28.99
Urban	245	71.01
*Year of study*		
First year	96	27.83
Second year	156	45.22
Third year or higher	93	26.95
*Age at first sex*		
≤ 15 years	28	8.31
16–17 years	59	17.51
18+ years	250	74.18

### Regular partner's fidelity

Although the majority of respondents (87.06%) were either not sure (42.27%) or definitely knew that their regular sexual partners did not have any other sexual partner(s) during the term of their sexual relationship (44.79%), it appeared that a significant number of them (12.93%) knew that their regular sexual partners had other sexual relationships. In other words, 12.93% of the respondents knew about their regular partners’ infidelity during the period that they were having sex together. The findings also revealed that state university respondents had higher infidelity levels (13.88%) than their private university counterparts (9.72%). However, when these responses were analysed across gender, it appeared the gender differences had limited influence on the respondents' knowledge of regular partners' infidelity, as reflected by 12.97% and 12.88% of male and female respondents, respectively, who were definitely certain that their regular partners cheated on them. In support of this, the chi-square test results pointed out that the differences across universities and gender in their reports of regular partners' fidelity were not statistically significant, as reflected by the following statistics 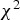 = 3.41 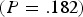 and 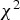 = 0.08 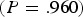 for the place of study and gender, respectively.

### Personal fidelity

The results showed that slightly above a third (34.39%) of the respondents consented to having had sexual intercourse with other partners while they were in sexual relationships with their regular partners (self-reported infidelity). Remarkable and encouraging was that 58.60% of respondents reported self-fidelity. The distribution of self-reported infidelity was fairly homogeneous across the two universities, although a slightly higher proportion of respondents at the private university (37.50%) reported personal infidelity than those from the state university (33.47%). However, these differences proved to be statistically insignificant as shown by 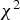 = 1.34 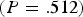. As expected, more males (38.25%) than females (29.01%) reported personal infidelity. The differences in reported personal infidelity between male and female respondents were in fact statistically significant at 5% as reflected by 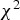 = 7.98 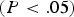. This means that the reported differences in personal infidelity between male and female respondents were real, implying that more males were likely to report personal infidelity than their female counterparts.

### Risk perception from regular partner's sexual behaviour

Above a quarter of the sexually active respondents (27.13%) felt at risk of contracting sexually transmitted infections (STIs) due to their regular partners’ sexual behaviours. Less than half (44.16%) of the respondents were definitely sure that the sexual behaviours of their regular partners did not expose them to the risk of contracting HIV. A larger proportion of respondents from the state university felt at risk of acquiring STIs from their regular partners' sexual practices than those from the private university (29.39% versus 19.44%). The proportions of respondents who did not feel at risk due to their partners' behaviours were similar across the two universities, though those from the private university were slightly higher (45.83%) than those from the state university (43.67%). Across gender, more male respondents (30.81%) than female respondents (21.97%) felt at risk. This may be explained by the apparent risky sexual behaviours often exhibited by female students at universities. A greater proportion of females than males were content with the sexual behaviours of their regular partners (51.52% versus 38.92%, respectively).

These differences across universities and gender were, however, statistically insignificant at 5% level of significance as reflected by 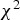 = 3.26 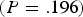 and 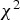 = 5.37 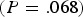, respectively, for the place of study and gender. It, therefore, means that state and private university respondents’ and male and female respondents' HIV risk perceptions from their regular partners' sexual behaviours are similar to each other. Table 2 shows the results discussed above.

**Table 2.** Summary of risk perception from regular partner's sexual behaviour.

Respondents	*n*	Definitely yes	Not sure	Definitely no		*P*-value
*Place of study*						
State university	245	72 (29.39)	66 (26.94)	107 (43.67)	3.26	.196
Private university	72	14 (19.44)	25 (34.72)	33 (45.83)		
*Gender*						
Male	185	57 (30.81)	56 (30.27)	72 (38.92)	5.37	.068
Female	132	29 (21.97)	35 (26.52)	68 (51.52)		
Overall	317	86 (27.13)	91 (28.71)	140 (44.16)		

Note: Figures in parentheses are the percentages, while those outside are the frequencies.

### Risk perception from casual partner's sexual behaviour

The majority of respondents (52.26%) felt at increased risk of contracting STIs as a result of their casual partner's sexual behaviours. Only 17.09% of respondents were content with the behaviour of their casual partners as far as the risk of acquiring STIs was concerned. More state university respondents (55.19%) felt that the sexual behaviour of their casual partners exposed them to the risk of STIs than those from the private university (42.22%). This means that the risk perceptions of state university respondents due to their casual partners' behaviour were fairly higher than those possessed by their private university counterparts. More males (55.80%) than females (44.26%) felt at risk of acquiring STIs due to their casual partners' sexual behaviours, whereas more female respondents as compared to male respondents (21.31% versus 15.22%) were comfortable with the sexual behaviour of their sexual partners. However, these differences were also not statistically significant as shown by 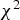 = 2.37 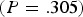 and 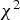 = 2.40 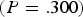 for the place of study and gender, respectively. These results are presented in Table 3.

**Table 3.** Summary of risk perception from casual partner's sexual behaviour.

Respondents	*n*	Definitely yes	Not sure	Definitely no		*P*-value
*Place of study*						
State university	154	85 (55.19)	44 (28.57)	25 (16.23)	2.37	0.305
Private university	45	19 (42.22)	17 (37.78)	9 (20.00)		
*Gender*						
Male	138	77 (55.80)	40 (28.99)	21 (15.22)	2.40	0.300
Female	61	27 (44.26)	21 (34.43)	13 (21.31)		
Overall	199	104 (52.26)	61 (30.65)	34 (17.09)		

Note: Figures in parentheses are the percentages, while those outside are the frequencies.

### Risk perception from own sexual behaviour

Results revealed that about a third of the respondents (32.43%) felt that their own sexual behaviours were exposing them to the risk of contracting HIV. However, it was not clear why they continued to engage in activities that so much exposed them to HIV risk, given that they were fully aware of the risk posed by their actions. Less than half of the respondents (41.14%) felt that their own behaviours were safe and did not expose them to the risk of HIV infections. This is alarming in this age of HIV, since it means that 58.86% of the sexually active respondents were either aware or unaware that their sexual behaviours were exposing them to HIV risk. A slightly higher proportion of respondents from the state university (35.38%) revealed that they felt exposed to the risk of HIV transmission due to their own sexual behaviours as compared to only 21.92% from the private university. This difference in results across universities was real and statistically significant at 1% as shown by 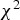 = 6.73 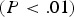. This means that more respondents from the state university felt at risk due to their own sexual behaviours than those from the private university. Table 4 shows respondents' HIV risk perceptions that emanate from their own sexual behaviours.

**Table 4.** Summary of risk perception from own sexual behaviour.

Respondents	*n*	Definitely yes	Not sure	Definitely no		*P*-value
*Place of study*						
State university	260	92 (35.38)	70 (26.92)	98 (37.69)	6.73**	<.001
Private university	73	16 (21.92)	18 (24.66)	39 (53.42)		
*Gender*						
Male	199	79 (39.70)	56 (28.14)	64 (32.16)	18.29**	<.001
Female	134	29 (21.64)	32 (23.88)	73 (54.48)		
Overall	333	108 (32.43)	88 (26.43)	137 (41.14)		

Note: Figures in brackets are the percentages, while those outside are the frequencies.

**Statistical significance at 1%.

In addition, more males than females acknowledged the risk of HIV posed by their own behaviours, as reflected by 39.70% and 21.64%, respectively, for males and females. This was reinforced by the observation that 54.48% of sexually active female respondents were definitely sure that their own behaviours were not exposing them to the risk of HIV infections, while only 32.16% of their male counterparts were certain that their behaviours were safe and did not expose them to the risk of HIV. Also startling to note was a sizeable percentage of respondents (26.43%) who were not sure or aware about the risk (or otherwise) posed by their own sexual behaviours. Chi-square test results of 18.29 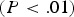 invariably confirmed the differences in HIV risk perceptions from own behaviour between male and female respondents. These findings strengthen the argument that more males than females are more afraid that their own sexual behaviours might land them with HIV. Refer to Table 4 for these results.

### Chances of HIV infection

It emerged that almost two-thirds of the sexually active respondents (66.56%) had thought of their chances of contracting HIV prior to the conduct of this study. These findings may seem encouraging, but the fact that 33.44% of the sexually active students had never thought of their chances of contracting HIV is a cause for concern, especially given the nature of relationships that most students normally enter into. Between the two universities, it emerged that 67.66% of students from the state university and 62.86% from the private university had thought about the HIV risk they face. However, these differences were not statistically significant, 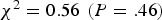. Similarly, female and male respondents had fairly similar proportions of those who had thought of their chances of contracting HIV (68.75% versus 64.97%). These were also not statistically different from each other, 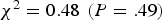.

Most sexually active respondents (71.38%) revealed that their chances of contracting HIV were either low (53.77%) or not there at all (17.61%). Only 28.62% of them felt that their chances of contracting HIV were either medium (21.70%) or high (6.92%). This clearly shows that respondents had very low personal HIV risk perception. This pattern was fairly similar across the two universities, although it appeared that state university students were slightly sceptical about their chances of contracting HIV than their private university counterparts. There were no statistically significant differences between respondents from the two universities in terms of their personal risk perceptions, as shown by a chi-square statistic of 5.41 (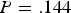). This implies that the differences in personal HIV risk perceptions among respondents from the two universities were not different from zero.

Correspondingly, more male (33.69%) than female respondents (21.37%) classified their chances of contracting HIV as either medium or high. This difference was supported by a chi-square statistic of 7.63 (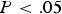), which showed that there was a real difference in personal HIV risk perceptions held by male and female respondents. This may be due to highly risky sexual activities that are more common among male students, such as having many sexual partners and engaging in sex under the influence of alcohol.

### Risk perceptions of fellow students

Most of the sexually active respondents (89.49%) described their fellows' sexual behaviours as either risky or very risky. Only a tenth (10.51%) felt that their fellows' sexual behaviours were either safe or very safe. Although slightly more female respondents (90.08%) reported that their colleagues' sexual behaviours were at least risky than male respondents (89.08%), the difference in HIV risk perceptions of students about their fellows' sexual behaviours across gender was statistically insignificant, as shown by a chi-square statistic of 4.86 (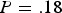). In other words, the difference in views between male and female respondents was statistically different from zero.

Across the two universities, somewhat more respondents from the state university (90.79%) than those from the private university (85.07%) viewed their fellows' sexual behaviours as at least risky. In other words, only 9.21% of the state university respondents viewed their fellows' behaviour as either safe or very safe as compared to almost double that figure (14.93%) for private university respondents who had such perceptions about their fellows. A chi-square statistic of 9.27 (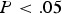) means that the difference in respondents' perceptions of their fellows' sexual behaviours at the two universities was statistically significant at 5%. This means that the HIV risk perceptions of fellow students held by respondents from the state university were different from those held by respondents from the private university.

## Discussions

This article sought to ascertain the personal HIV risk perceptions of university students in Zimbabwe and to explore their knowledge of possible sources of HIV risk. In addition, it envisaged establishing the predictors of personal HIV risk perceptions of students. Results revealed that the level of infidelity was high among the sexually active students, with a sizeable number of them acknowledging either their own infidelity or that of their regular sexual partners. These results underscore the prevalence of multiple concurrent partnerships and the ‘no strings attached’ types of sexual relationships among the sexually active university students. The other factors that may be promoting such infidelity among students were what some respondents referred to as ‘friends with benefits’, a situation where friends can have sexual intercourse, with full knowledge that they both have their ‘proper’ sexual partners. The findings were consistent with various previous studies on university students (Heeren, Jemmott, Mandeya & Tyler 2007; Maharaj & Cleland 2008; Njikam nee Savage 2005; Nshindano & Maharaj 2008; Ntata *et al*. 2008; Peltzer 2005). An intriguing question would be to know why students continue in those sexual relationships when they are fully aware that their regular partners are having other sexual affairs, especially in this era of HIV. This could possibly be because other students may be entering into sexual relationships without full knowledge that their partners have other relationships or that they know but want to solicit some benefits from these extra partners, such as money or gifts. Such multiple sexual relationships are very conducive to the spread of STIs, especially HIV. The findings that more males than females reported personal infidelity were in line with the socially acceptable norms that justify and perpetuate male infidelity and discourage or punish similar acts when perpetrated by females. This may, thus, point to the far-reaching influence of cultural norms in shaping the sexual behaviour of university students.

Despite their awareness of infidelity of their regular sexual partners and also their own, a sizeable proportion of respondents did not feel at risk of contracting HIV. In other words, regular sexual partners were not viewed as important HIV risk factors, as close to a half of the respondents were not threatened by the behaviour of their regular sexual partners. However, they felt much exposed to the risk of HIV due to the behaviour of their casual sexual partners. This was possibly due to the non-committal nature of such relationships, which in many instances may provide fertile ground for the transmission of STIs, including HIV, in the form of one night stands and no-strings-attached relationships. The findings are consistent with results from previous studies that revealed that HIV-preventative methods, such as condoms, were not popular among regular sexual partners, reflecting that people normally do not view them as a source of HIV risk (Adetunji 2000; Prata, Vahidnia & Fraser 2005).

Consistent with findings from previous studies, personal HIV risk perceptions of university students were found to be very low (Ferrer *et al*. 2007; Maswanya *et al*. 1999; Nshindano & Maharaj 2008; Ntata *et al*. 2008; Peltzer 2005; Rahamfey *et al*. 2008). This was in spite of their acknowledgement that they engaged in a variety of risky sexual behaviours, such as having multiple sexual partners. These findings clearly show that university students are not personally worried about getting HIV. The low HIV risk perceptions of students are quite difficult to understand given that most students are familiar with the risky environment that characterises universities. It appeared that information fatigue was creeping into this group of young people as they have heard about HIV over and over again. It also appeared that students downplay their exposures to HIV risks, which may possibly reflect the inherent stigmatisation associated with the disease. As such, ‘denial and distancing’ may be the factor behind low personal HIV risk perception (Brown *et al*. 2000). Furthermore, the low personal HIV risk perceptions of students may underscore that they have already taken some steps to limit their exposures, especially through the use of condoms. The belief in the efficacy of condoms may entail that students may engage in sexual activities, even with multiple partners, yet they may not feel at high risk of contracting HIV. Nevertheless, these findings were contrary to those reported by Omungo (2008) and Njikam nee Savage (2005) who found high personal HIV risk perceptions among students from Kenyan and Cameroonian universities (Njikam nee Savage 2005; Omungo 2008).

Personal HIV risk perception is key in the pursuit of sexual behaviour change. However, the study found that over 40% of the students had never thought of their chances of being infected with HIV. This was very disturbing, especially in Zimbabwe where the HIV epidemic is generalised. It may reflect the fact that a significant number of respondents might be seeing themselves as immune from HIV or thinking that it was other people's problem and not theirs. These findings are slightly better than those reported for South African university students, where 83% of the sampled students had never been worried about their risk of contracting HIV (Peltzer 2005).

Respondents' perceptions of HIV risk for fellow students were surprisingly high. These findings may underscore the prevalence of risky sexual behaviours among the student population and support the argument that universities are sexually permissive environments that may fuel the spread of HIV (Adefuye *et al*. 2009; Masvaure *et al*. 2009; Omungo 2008; Sabone *et al*. 2007). While this was expected, it was in sharp contrast with their personal HIV risk perceptions, which were very low. However, what remains unclear is why the majority would view the sexual behaviours of their fellows as risky, while they viewed theirs as safe. This may substantiate the claim that university students may often downplay their HIV risk exposures, but are comfortable to report those of their fellows, which may then reflect their own. The higher perception of HIV risk of fellows among state university students than among the private university students may underline that the state university environment is relatively risky as compared to the private university, possibly due to lack of administrative prohibitions at the state university as compared to the private university. Higher risk perception of HIV risk of fellows among female students than male respondents may be testimony to the wider sexual network of female students than that of their male counterparts who are often financially constrained to finance numerous sexual partners.

## Conclusions

The complexity of the HIV epidemic calls for young people to be at the centre of its prevention. The findings of this study draw attention to the low personal HIV risk perceptions of university students in Zimbabwe. Although they reported risky sexual behaviours of their fellow students, the majority appeared personally unmoved by the risk of HIV infections. Interventions should, therefore, focus on raising personal awareness of the risk of HIV and equip students with ways of correctly assessing their own HIV risk exposures, so as to stimulate preventative measures that curtail the spread of HIV among this group of young people. In addition, issues of stigmatisation and discrimination should be addressed to encourage students to come out in the open about their HIV status. This would go a long way in heightening personal HIV risk perceptions of university students. While administrative prohibitions are often unwelcome among students, the state university can borrow a leaf from the private university and restrict some of the practices that promote sex among their students, thereby cutting down the students' risk exposures.
